# Novel elucidation and treatment of pancreatic chronic graft-versus-host disease in mice

**DOI:** 10.1098/rsos.181067

**Published:** 2018-10-17

**Authors:** Shin Mukai, Yoko Ogawa, Fumihiko Urano, Yutaka Kawakami, Kazuo Tsubota

**Affiliations:** 1 Department of Ophthalmology, Keio University School of Medicine, 35 Shinanomachi, Shinjuku-ku, Tokyo 160-8582, Japan; 2 Department of Medicine, Division of Endocrinology, Metabolism, and Lipid Research, Washington University School of Medicine, St Louis, MO, USA; 3 Department of Pathology and Immunology, Washington University School of Medicine, St Louis, MO, USA; 4 Division of Cellular Signalling, Institute for Advanced Medical Research, Keio University School of Medicine, Tokyo, Japan

**Keywords:** pancreatic graft-versus-host disease, endoplasmic reticulum stress, 4-phenylbutyric acid, inflammation, fibrosis

## Abstract

Chronic graft-versus-host disease (cGVHD) is a severe complication of allogeneic haematopoietic stem cell transplantation. There is a growing understanding of cGVHD, and several effective therapies for cGVHD have been reported. However, pancreatic cGVHD is a potentially untapped study field. Our thought-provoking study using a mouse model of cGVHD suggested that the pancreas could be impaired by cGVHD-induced inflammation and fibrosis and that endoplasmic reticulum (ER) stress was augmented in the pancreas affected by cGVHD. These findings urged us to treat pancreatic cGVHD through reduction of ER stress, and we used 4-phenylbutyric acid (PBA) as an ER stress reducer. A series of experiments has indicated that PBA can suppress cGVHD-elicited ER stress in the pancreas and accordingly alleviate pancreatic cGVHD. Furthermore, we focused on a correlation between epithelial to mesenchymal transition (EMT) and fibrosis in the cGVHD-affected pancreas, because EMT was conceivably implicated in various fibrosis-associated diseases. Our investigation has suggested that the expression of EMT markers was increased in the cGVHD-disordered pancreas and that it could be reduced by PBA. Taken together, we have provided a clue to elucidate the pathogenic process of pancreatic cGVHD and created a potentially effective treatment of this disease using the ER stress alleviator PBA.

## 1. Introduction

Chronic graft-versus-host disease (cGVHD) is an immunologically mediated complication and hampers success in allogeneic haematopoietic stem cell transplantation (HSCT). In medical settings, cGVHD typically occurs in allogeneic HSCT recipients six months or later after transplantation, and they can suffer from multisystem disorders which resemble those induced by autoimmune diseases [1,2]. For instance, dry eye, skin rashes, diarrhoea and respiratory failure are caused by cGVHD, and these disabling symptoms can impact detrimentally on patients' quality of life [1]. From a scientific point of view, it is conceivable that cGVHD arises from minor histocompatibility differences between donor-derived immune cells and recipient cells [1]. Although cGVHD has been studied intensively over a long period of time, the exact mechanisms of cGVHD remain to be unravelled. However, there is increasing elucidation of the pathogenic process of cGVHD. Most recently, several research groups have used a mouse model of cGVHD and developed potentially efficacious methods to cure cGVHD in various organs [3–7]. Despite the recent advancement of therapies for cGVHD, to the best of our knowledge, no comprehensive biological investigation into pancreatic cGVHD has been reported thus far. This fact urged us to gain more insights into cGVHD in the pancreas. In clinical practice, cGVHD patients can be affected by diabetes, and it has been conceived to arise from corticosteroid therapies, not from disorders in the pancreas [8,9]. Thus, we envisaged that it would be of great medical importance to focus on the state of the pancreas in cGVHD sufferers.

Our recent work has demonstrated (i) that endoplasmic reticulum (ER) stress is augmented in organs impaired by cGVHD, (ii) that the augmentation of ER stress is detrimentally associated with the development of inflammation and fibrosis caused by cGVHD and (iii) that the attenuation of ER stress by systemic injection of 4-phenylbutyric acid (PBA) could be a safe, reliable and robust strategy to pave the way for the treatment of clinical cGVHD [10].

ER stress is conceived to be connected with chronic inflammation and age-related disorders [11,12]. The ER is a cellular organelle which maintains the proper functions of each cell. After the synthesis of proteins in the ER, they are required to be folded into adequate forms, and the protein folding can be achieved with the assistance of ER chaperones [13]. However, the essential functions of the ER can be disturbed by the following phenomena: hypoxia, calcium ion depletion, oxidative injury and viral infections [14,15]. Aberrant accumulation of unfolded and/or misfolded proteins causes ER stress, and accordingly, the following three transmembrane proteins are released from glucose-regulated protein 78 (GRP78) to start the unfolded protein response (UPR): inositol requiring (IRE) 1*α*, PKR-like ER kinase (PERK) and activating transcription factor 6*α* [13]. However, the prolonged and/or unsuccessful UPR causes the activation of inflammatory and apoptotic pathways [13]. Hence, in the case where the UPR is prolonged and/or unsuccessful, it results in the out-of-control expression and/or activation of (i) the proinflammatory molecules transcription factor nuclear factor kappa-light-chain-enhancer of activated B cells (NF-κB) and thioredoxin interaction protein (TXNIP) and (ii) the apoptotic protein C/EBP homologous protein (CHOP) [16–21].

In this study using a mouse model of cGVHD, it was envisioned that the pancreas could be susceptible to cGVHD-elicited inflammation and fibrosis and that the use of ER stress reducer PBA could be a promising approach to cure pancreatic cGVHD. Herein, we report our novel endeavours to unravel the pathogenic process of cGVHD in the pancreas and treat the potentially under-explored disease.

## 2. Material and methods

Eight-week-old B10.D2 and BALB/c mice were purchased from Sankyo Laboratory, Inc. (Tokyo, Japan). All the scientific experiments on mice were performed in accordance with the Animal Welfare Act at Keio University School of Medicine. Our protocols for experiments on animals were approved by the animal care and use committee at Keio University (Approval no. 09152). It should also be noted that no fieldwork was conducted in this study.

### 2.1. Bone marrow transplantation

Bone marrow transplantation (BMT) was carried out to afford a murine model of cGVHD [22]. In the case where the donors were B10.D2 mice and the recipients were BALB/c mice, it was allogeneic BMT (allo-BMT) to produce a murine model of cGVHD. By contrast, BMT from BALB/c to BALB/c mice was syngeneic BMT (syn-BMT), and therefore cGVHD did not occur in the transplant recipients. The recipient mice without cGVHD served as syngeneic control subjects. The recipients were irradiated with 700 cGy prior to the BMT, and the lethal irradiation was performed using a Gammacel 137 Cs source (Hitachi Medico, Ltd, Tokyo, Japan). A suspension containing 1 × 10^6^ bone marrow cells and 2 × 10^6^ spleen cells from the donors was administered to each of the recipient mice via tail vein. The donor cells were suspended in RPMI 1640 (Life Technologies Japan Ltd, Tokyo, Japan).

### 2.2. Treatment of allogeneic bone marrow transplantation recipient mice with 4-phenylbutyric acid

We conducted BMT as described above, and the allo-BMT recipient mice were divided into two groups. One group was treated with PBA (10 mg kg^−1^) (Aldrich, St Louis, MO, USA), and the other was given the solvent-vehicle phosphate buffer saline (PBS; pH 7.4) by intraperitoneal injection. We administered the inhibitor or the solvent-vehicle to the allo-BMT recipients once per day from Day 10 to Day 27 after BMT. They were sacrificed Day 28 after BMT.

### 2.3. Histological analysis of the pancreas and immunohistochemistry for paraffin-embedded tissue sections

Three or four weeks after BMT, the pancreas was collected from the transplant recipient mice. These samples were subsequently fixed with 10% neutral-buffered formalin and embedded in paraffin. The paraffin blocks were cut into 7 µm-thick sections, and then stained with (i) haematoxylin and eosin (HE), (ii) Mallory's trichrome [23,24] and (iii) antibodies used in this study. For the immunohistochemical assays, paraffin was removed in the first instance. To stain the sections with a CD45 antibody (30-F11, BD Pharmingen, San Jose, CA, USA) or an E-cadherin antibody (24e10, Cell Signaling Technology, Danvers, MA, USA), they were immersed in the antigen retrieval solution (Target Retrieval Solution; Dako, Glostrup, Denmark) and then boiled with a microwave oven for 10 min, and the reactions between the antigens in tissue sections and the primary antibodies were conducted at 4°C overnight. The sections were then treated with fluorophore-labelled secondary antibodies with 4′,6-diamidino-2-phenylindole (DAPI) for nuclear staining at room temperature for 45 min and mounted with an anti-fading mounting medium (Fluorescent Mounting Medium; Dako). Fluorescence images were taken with an LSM confocal microscope (Carl Zeiss, Jena, Germany). As for the counting of CD45^+^ cells, five areas of each tissue section were randomly photographed at 200× magnification, and the number of CD45^+^ cells in the individual images was subsequently determined. With respect to secondary antibodies, goat anti-rat IgG (H + L) secondary antibody, Alexa Fluor 568 conjugate (Molecular Probes) and goat anti-rabbit IgG (H + L) secondary antibody, Alexa Fluor 488 conjugate (Molecular Probes) were used to detect CD45. Rat IgG2b, *κ* (eB149/10H5, eBioscience, San Diego, CA, USA) and rabbit IgG (Cell Signaling Technology, Danvers, MA, USA) were used as isotype controls for CD45 and E-cadherin, respectively.

### 2.4. Immunohistochemistry for frozen tissue sections

Three or four weeks after BMT, the pancreas was collected from the transplant recipient mice. These samples were subsequently fixed with 10% neutral-buffered formalin and embedded in Tissue-Tek OCT Compound (Sakura Finetek, Torrance, CA, USA) to produce formalin-fixed frozen blocks. The frozen blocks were then cut into 7 µm-thick sections and preserved at −80°C until they were used.

In order to carry out multiple staining for CHOP (H-43, Santa Cruz Biotechnology, Santa Cruz, CA, USA) and CD68 (FA-11, AbD Serotec, Kidlington, UK), the formalin-fixed frozen sections were defrosted at 37°C, fixed with acetone at room temperature for 20 min, washed with PBS (3 × 3 min) and heated up in the antigen retrieval solution (HistoVT One; Nakalai Tesque, Kyoto, Japan) at 70°C for 20 min with a water bath.

After the activity of the target antigens was recovered, the sections were blocked with 10% normal goat serum, and the reactions between the antigens in tissue sections and the primary antibodies were conducted at 4°C overnight. The sections were then treated with fluorophore-labelled secondary antibodies with DAPI for nuclear staining at room temperature for 45 min and mounted with an anti-fading mounting medium (Fluorescent Mounting Medium; Dako). Fluorescence images were taken with an LSM confocal microscope (Carl Zeiss, Jena, Germany). In this immunohistochemical examination, the following secondary antibodies were used: goat anti-rabbit IgG (H + L) secondary antibody, Alexa Fluor 488 conjugate (Molecular Probes, Eugene, OR, USA) goat anti-rat IgG (H + L) secondary antibody, Alexa Fluor 568 conjugate (Molecular Probes) and goat anti-American hamster IgG (H + L) secondary antibody, Alexa Fluor 568 conjugate (Molecular Probes)*.* With regard to isotype controls, rat IgG2a (54447, R&D Systems, Minneapolis, MN, USA) and rabbit IgG (Cell Signaling Technology) were used for CD68 and CHOP, respectively.

### 2.5. Electron microscopy

Transmission electron microscopic analysis was performed according to standard protocols. Tissues were collected from the murine pancreas, immediately fixed with 2.5% glutaraldehyde in 0.1 M phosphate buffer (pH 7.4) at 4°C for 4 h and washed three times with 0.1 M phosphate buffer. The samples were subsequently fixed again with 2% osmium tetroxide, dehydrated in a graded series of ethanol and 100% propylene oxide, and embedded in epoxy resin. One micrometre sections were made from the processed tissues and then stained with methylene blue. The thick sections were observed with a microscope to find parts which were suitable for preparation of ultrathin sections. The obtained sections were placed on mesh grids, stained with uranylacetate and lead citrate and examined with an electron microscope (1230 EXII; JEOL, Tokyo, Japan). All electron micrographs were acquired with a bio scan camera (Gatan bio scan camera model 792, Tokyo, Japan).

### 2.6. Immunoblotting analysis

The tissues of interest were placed in Eppendorf tubes, and pre-cooled RIPA buffer was added to the tubes. The tissues were then homogenized using an electric homogenizer. After the samples were on ice for 1 h, they were centrifuged at 15 000 r.p.m. at 4°C for 5 min. The supernatants were subsequently collected in fresh tubes on ice and used as cell lysates. An equal amount of 5× Laemmli buffer was added to each cell lysate, followed by protein denaturation at 100°C for 5 min. Equal amounts of protein from each sample were loaded into the wells of SDS-PAGE gels and then resolved. The proteins were transferred from the gels to membranes at 15 V for 20 min. The membranes were blocked with 5% skim milk or 5% BSA in 1 × TBST (a mixture of Tris-buffered saline and tween 20) at room temperature for 1 h. The membranes were then incubated with primary antibodies at 4°C overnight. The primary antibodies were diluted 1000 times with 5% skim milk or 5% BSA in 1 × TBST. After the primary antibody incubation, the membranes were washed with 1 × TBST (3 × 10 min), subjected to secondary antibody at room temperature for 1 h and then washed with 1 × TBST (3 × 10 min) and 1 × TBS (2 × 10 min). The proteins of interest were visualized using either of the following two methods. (i) Colorimetric detection of the target proteins was conducted using BCIP/NBT substrate (Promega, WI, USA). (ii) Signals were developed with an enhanced chemoluminescence (ECL) detection reagent (GE Healthcare, Littlecalfont, UK), and the target proteins were subsequently visualized with a LAS 4000 mini chemiluminescence imaging system (Fujifilm/GE Healthcare). Densitometric analysis of the obtained protein bands was conducted with the use of the image processing software ImageJ. The primary antibodies used in this experiment were as follows: GRP78 (Abcam, Cambridge, UK), phospho-PERK (Thr980, Cell Signaling Technology), PERK (C33E10, Cell Signaling Technology), phosphor-IRE1*α* (Thermo Fisher Scientific, Waltham, MA, USA), IRE1*α* (14C10 Cell Signaling Technology), phosphor-eIF2*α* (119A11, Cell Signaling Technology), eIF2*α* (Cell Signaling Technology), CHOP (9C8, Thermo Fisher Scientific), TXNIP (D5F3E, Cell Signaling Technology), NF-κB (Abcam), IL-6 (Abcam), connective tissue growth factor (CTGF) (Abcam), E-cadherin (24e10, Cell Signaling Technology), α-smooth muscle actin (SMA) (1A4, Abcam), Snail (C15D3, Cell Signaling Technology) and β-actin (AC-15, Abcam). With regard to the secondary antibodies, (i) when the protein bands were visualized by developing a colour, either an AP-conjugated anti-mouse IgG antibody (Promega) or an AP-conjugated anti-rabbit IgG antibody (Promega) was used, and (ii) either an HRP-conjugated anti-mouse antibody (Thermo Fisher Scientific) or an HRP-conjugated anti-rabbit antibody (Thermo Fisher Scientific) was required to detect the target proteins by ECL.

### 2.7. Measurement of blood glucose levels

Blood glucose levels were measured by the Japanese company Sanritsu Zelkova (Kanagawa, Japan). The measurement was conducted with the use of a kit (27E1X80166000006, LSI Medience, Japan).

### 2.8. Statistical analysis

Statistical significance was determined with the use of unpaired Student's *t*-test. Differences are considered significant in the case of *p* < 0.05. The acquired data are presented as means ± s.d.

## 3. Results

### 3.1. Histological analysis of the pancreas affected by chronic graft-versus-host disease

**Figure 1.**
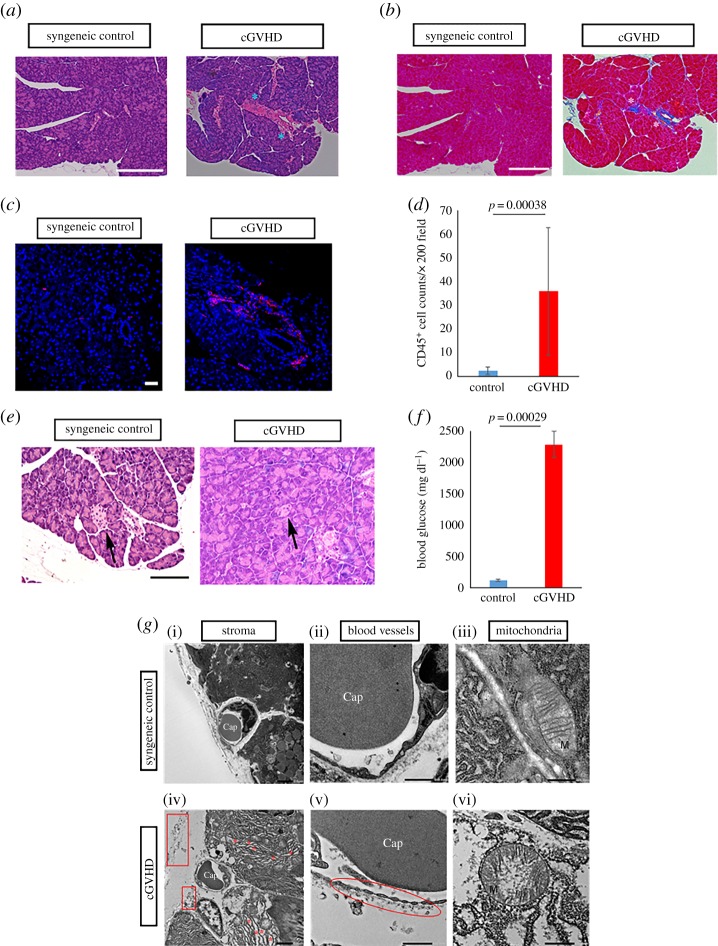
Histological assays for the pancreas disordered by cGVHD. (*a*) HE pictures of the cGVHD-affected pancreas and its syngeneic control partner. The images were taken at 200× magnification, and the scale bar is 200 µm. Severely inflamed portions are shown with blue asterisks. The enlarged version of each picture is shown in electronic supplementary material, figure S1. (*b*) Mallory's staining for the cGVHD-disordered pancreas and its syngeneic control equivalent. The photographs were taken at 200× magnification, and the scale bar is 200 µm. Aberrantly fibrotic areas are shown with white asterisks. The enlarged version of each picture is shown in electronic supplementary material, figure S2. (*c*) Immunostaining for the generic leucocyte marker CD45 in the cGVHD-impaired pancreas and its syngeneic control counterpart. Cell membranes and nuclei are stained red and blue, respectively. The images were taken at 200× magnification, and the scale bar is 20 µm. The enlarged version of each picture is shown in electronic supplementary material, figure S3. (*d*) The density of CD45 positive cells in the pancreas from syn-BMT recipient mice (blue) and cGVHD-affected ones (red). The data are presented as means ± s.d., control: *n* = 3, cGVHD: *n* = 3. (*e*) HE pictures of pancreatic islets in syn-BMT recipient mice and cGVHD-disordered ones. A pancreatic islet is shown with a black arrow. The images were taken at 400× magnification, and the scale bar is 100 µm. The enlarged version of each picture is shown in electronic supplementary material, figure S4. (*f*) Blood glucose levels in syn-BMT recipient mice and cGVHD-disordered ones. The data are presented as means ± s.d., control: *n* = 5, cGVHD: *n* = 5. (*g*) Electron micrographs of cGVHD-affected pancreas and its syngeneic control counterpart. Cap, capillary; M, mitochondrion. The pictures of stroma in the pancreas (i,iv) were at 2000× magnification, and the scale bar is 5 µm. Asterisks are placed where the ER is expanded due to the accumulation of proteins, and cell debris is shown with rectangles. The photographs of blood vessels in the pancreas (ii,v) were at 5000× magnification, and the scale bar is 500 nm. Damaged blood vessels are displayed with an ellipse. The photographs of mitochondria in the pancreas (iii,vi) were at 15 000× magnification, and the scale bar is 500 nm. The enlarged version of each picture is shown in electronic supplementary material, figure S7.

In the first instance, we conducted histological investigation into the pancreas. As indicated by HE and Mallory's staining, tissues around ducts in the pancreas collected from allo-BMT recipients were excessively inflamed in association with mononuclear cell infiltration and fibrotic in concert with extracellular matrix accumulation (figure 1*a,b*; electronic supplementary material, figures S1 and S2). Conversely, when mice underwent syn-BMT, their pancreas seemed to be virtually normal (figure 1*a*,*b*; electronic supplementary material, figures S1 and S2). This finding indicated that pancreatic exocrine failure could be induced by cGVHD. Immunostaining for the generic leucocyte marker CD45 also revealed that abnormal immune cell migration was observed in the cGVHD-disordered pancreas by contrast with its syngeneic control equivalent (*p* = 0.00038) (figure 1*c*,*d*; electronic supplementary material, figure S3). In addition, the size of pancreatic islets was greatly reduced in the cGVHD-affected mice in contrast to that in their syngeneic control counterparts (figure 1*e*; electronic supplementary material, figure S4). Presumably owing to the dysfunctional exocrine and endocrine glands [25–27], the blood glucose levels in the cGVHD-affected mice were substantially greater than those in the syngeneic control subjects (*p* = 0.000029) (figure 1*f*). The degree of inflammation and fibrosis was determined by immunoblot assays for the inflammatory markers IL-6 and CTGF [28,29]. Our data indicated that these two markers were expressed at higher level in the cGVHD-affected pancreas compared with its syngeneic control partner (IL-6: *p* = 6.9 × 10^−5^, CTGF: *p* = 1.2 × 10^−4^) (electronic supplementary material, figures S5 and S6). Furthermore, electron micrographic analysis was carried out to examine the state of the pancreas more closely. The electron micrographs of cGVHD-affected pancreatic epithelia suggested (i) that the ER was abnormally expanded and distorted owing to aberrant accumulation of proteins, (ii) that the walls of blood vessels in the stroma were extremely thin in association with multiple fenestrations, (iii) that the cristae of mitochondria in acinar cells were distorted and damaged and (iv) that the mitochondria were expanded (figure 1*g*; electronic supplementary material, figure S7). As shown by these findings, the pancreas is presumably prone to cGVHD-triggered inflammation and fibrosis.

### 3.2. Elevation of endoplasmic reticulum stress markers in the pancreas disordered by chronic graft-versus-host disease

**Figure 2.**
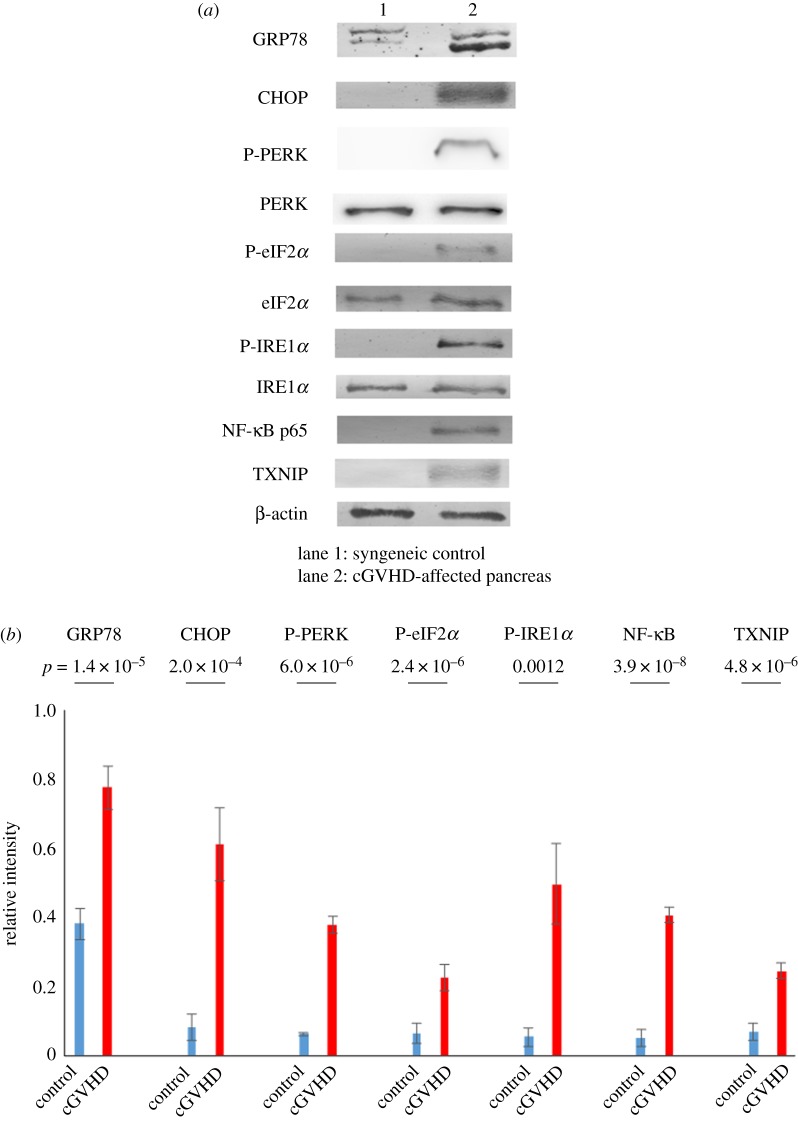
ER stress markers in the cGVHD-affected pancreas. (*a*) Immunoblot analysis of ER stress indicators and the associated inflammatory molecules was carried out (lane 1: syngeneic control subject, lane 2: cGVHD-affected pancreas). Cropped blots are displayed, and the corresponding full-length gels are shown in electronic supplementary material, figure S8. (*b*) The corresponding quantitative analysis of the protein bands was conducted. cGVHD-affected pancreas (red) and its syngeneic control counterpart (blue). Results are representative of two independently performed experiments with similar results. The data are presented as means ± s.d., control: *n* = 5, cGVHD: *n* = 5.

The novel histological elucidation of pancreatic cGVHD urged us to investigate a detrimental link between ER stress and cGVHD by measuring the following ER stress indicators: GRP78, CHOP, p-PERK, p-eIF2*α* and p-IRE1*α*. As judged by immunoblot analysis, the ER stress markers in the cGVHD-affected pancreas were expressed at higher level than its syngeneic control partner (GRP78: *p* = 1.3 × 10^−5^, CHOP: *p* = 2.0 × 10^−4^, p-PERK: *p* = 6.0 × 10^−6^, p-eIF2*α*: *p* = 2.4 × 10^−4^, p-IRE1*α*: *p* = 0.0012) (figure 2*a*,*b*; electronic supplementary material, figure S8*a–e*). As a consequence of the activation of ER stress signalling pathways, the following two inflammation-related molecules were elevated and/or activated in the cGVHD-disordered pancreas in contrast to its syngeneic control counterpart: NF-κB and TXNIP (NF-κB: *p* = 3.9 × 10^−7^, TXNIP: *p* = 4.9 × 10^−6^) (figure 2*a*,*b*; electronic supplementary material, figure S8*f,g*). These results are indicative of the fact that ER stress was elevated in the pancreas disordered by cGVHD.

### 3.3. Augmentation of epithelial to mesenchymal transition markers in the pancreas disordered by chronic graft-versus-host disease

**Figure 3.**
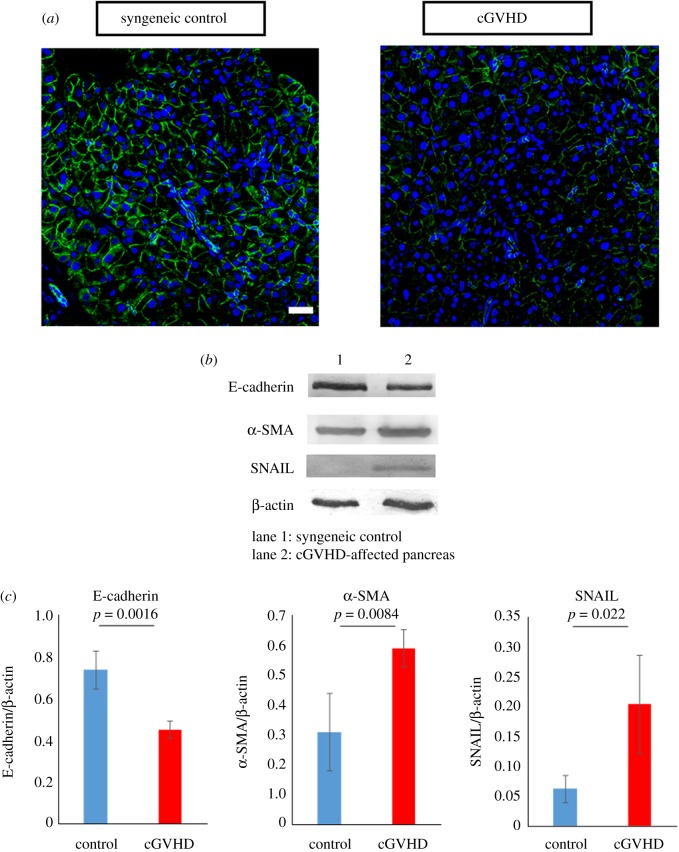
EMT markers in the cGVHD-affected pancreas. (*a*) Immunostaining for E-cadherin in cGVHD-affected pancreas and its syngeneic control counterpart. E-cadherin and cell nuclei are stained green and blue, respectively. The fluorescence images were at 200× magnification, and the scale bar is 20 µm. (*b*) Immunoblot analysis of E-cadherin and α-SMA was carried out (lane1: syngeneic control subject, lane 2: cGVHD-affected pancreas). Cropped blots are displayed, and the corresponding full-length gels are shown in electronic supplementary material, figure S9. (*c*) The corresponding quantitative analysis of the protein bands was conducted. cGVHD-affected pancreas (red) and its syngeneic control counterpart (blue). Results are representative of two independently performed experiments with similar results. The data are presented as means ± s.d., control: *n* = 5, cGVHD: *n* = 5.

Our examination indicated that the pancreas was vulnerable to fibrosis elicited by cGVHD, and a survey of literature revealed that epithelial to mesenchymal transition (EMT) was involved in various fibrosis-associated disorders [30–34]. Hence, we hypothesized that it would be the case with pancreatic cGVHD. In order to investigate our hypothesis, immunostaining and immunoblot analysis were conducted to examine the expression levels of EMT markers. As shown by immunohistochemical and immunoblot assays, the expression of the EMT indicator E-cadherin in the cGVHD-affected pancreas was decreased in contrast to that in its syngeneic control equivalent (*p* = 0.0016) (figure 3*a*–*c*; electronic supplementary material, figure S9*a*). Moreover, immunoblot analysis indicated that the EMT markers α-SMA and Snail in the cGVHD-affected pancreas were expressed at higher level than those in their syngeneic control partners (α-SMA: *p* = 0.0084, Snail: *p* = 0.022) (figure 3*b*,*c*; electronic supplementary material, figure S9*b,c*). These data suggested that EMT was linked to cGVHD-elicited fibrosis in the pancreas.

### 3.4. Treatment of pancreatic chronic graft-versus-host disease through reduction of endoplasmic reticulum stress

**Figure 4.**
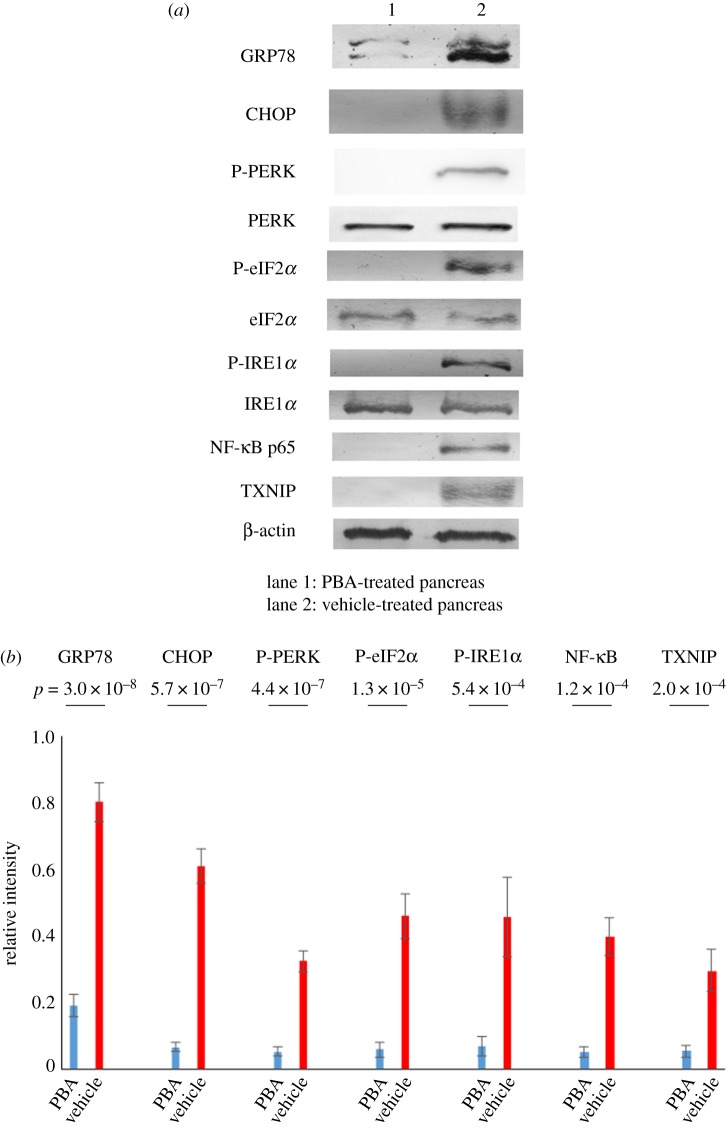
The ability of PBA to lower cGVHD-induced ER stress. (*a*) Immunoblot analysis of ER stress indicators and the associated inflammatory molecules was carried out (lane1: PBA-treated pancreas, lane 2: vehicle-treated pancreas). Cropped blots are displayed, and the corresponding full-length gels are shown in electronic supplementary material, figure S10. (*b*) The corresponding quantitative analysis of the protein bands was conducted. PBA-medicated pancreas (blue) and its vehicle-medicated counterpart (red). Results are representative of two independently performed experiments with similar results. The data are presented as means ± s.d., control: *n* = 6, cGVHD: *n* = 6.

Once we discovered the detrimental association between ER stress and pancreatic cGVHD, our ensuing attempt was to cure cGVHD in the pancreas by reducing ER stress. As described in the section Material and methods, we treated allo-BMT recipient mice with PBA or the solvent-vehicle. As judged by immunoblot analysis, the pancreas collected from the PBA-treated mice displayed the lower protein levels of GRP78, CHOP, p-PERK, p-eIF2*α* and p-IRE1*α* compared with that collected from their vehicle-treated counterparts (GRP78: *p* = 3.0 × 10^−8^, CHOP: *p* = 5.7 × 10^−7^, p-PERK: *p* = 4.4 × 10^−7^, p-eIF2*α*: *p* = 1.3 × 10^−5^, p-IRE1*α*: *p* = 5.4 × 10^−4^) (figure 4*a*,*b*; electronic supplementary material, figure S10*a*–*e*). As a consequence, the proinflammatory molecules NF-κB and TXNIP, which are in the downstream of ER stress signalling pathways, were repressed in the pancreas treated with PBA in comparison to its vehicle-treated equivalent (NF-κB: *p* = 1.2 × 10^−5^, TXNIP: *p* = 1.9 ^−^ 10^−4^) (figure 4*a*,*b*; electronic supplementary material, figure S10*f,g*).

### 3.5. Histological observations of chronic graft-versus-host disease target organs

**Figure 5.**
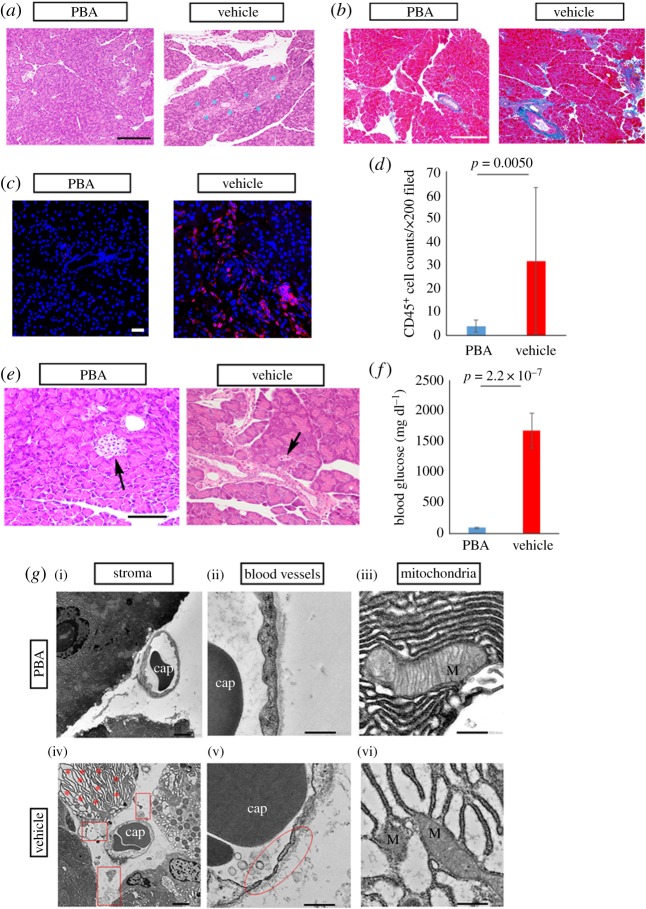
Effectiveness of PBA for cGVHD shown by histological investigation. (*a*) HE pictures of the PBA-injected pancreas and its vehicle-injected partner. The images were taken at 200× magnification, and the scale bar is 200 µm. Severely inflamed portions are shown with blue asterisks. The enlarged version of each picture is shown in electronic supplementary material, figure S11. (*b*) Mallory's staining for the pancreas treated with PBA and that treated with the solvent-vehicle. The photographs were taken at 200× magnification, and the scale bar is 200 µm. Aberrantly fibrotic areas are shown with white asterisks. The enlarged version of each picture is shown in electronic supplementary material, figure S12. (*c*) Immunostaining for the generic leucocyte marker CD45 in the PBA-medicated pancreas and its vehicle-medicated equivalent. Cell membranes and nuclei are stained red and blue, respectively. The images were taken at 200× magnification, and the scale bar is 20 µm. The enlarged version of each picture is shown in electronic supplementary material, figure S13. (*d*) The density of CD45 positive cells in the PBA-treated pancreas (blue) and its vehicle-treated counterpart (red). The data are presented as means ± s.d., PBA: *n* = 3, vehicle: *n* = 3. (*e*) HE pictures of pancreatic islets in PBA-medicated recipient mice and vehicle-medicated ones. A pancreatic islet is shown with a black arrow. The images were taken at 400× magnification, and the scale bar is 100 µm. The enlarged version of each picture is shown in electronic supplementary material, figure S15. (*f*) Blood glucose levels in PBA-treated mice and vehicle-treated ones. The data are presented as means ± s.d., PBA: *n* = 6, vehicle: *n* = 6. (*g*) Electron micrographs of the PBA-injected pancreas and its vehicle-injected counterpart. Cap, capillary; M, mitochondrion. The pictures of stroma in the pancreas (i,iv) were at 2000× magnification, and the scale bar is 5 µm. Asterisks are placed where the ER is expanded due to the accumulation of proteins, and cell debris is shown with rectangles. The photographs of blood vessels in the pancreas (ii,v) were at 5000× magnification, and the scale bar is 500 nm. Damaged blood vessels are displayed with an ellipse. The photographs of mitochondria in the pancreas (iii,vi) were at 15 000× magnification, and the scale bar is 500 nm. The enlarged version of each picture is shown in electronic supplementary material, figure S18.

In order to investigate the extent of inflammation and fibrosis in the PBA- and vehicle-treated pancreas from allo-BMT recipients, HE and Mallory staining was conducted. The HE and Mallory pictures suggested that areas around pancreatic ducts in PBA-medicated pancreas were less inflamed and fibrotic compared with its vehicle-medicated partner (figure 5*a*,*b*; electronic supplementary material, figures S11 and S12). In addition, immunohistochemical analysis showed (i) that the number of immune cells in the PBA-injected pancreas was vastly lower than that in its vehicle-injected equivalent (*p* = 0.005) (figure 5*c*,*d*; electronic supplementary material, figure S13) and (ii) that macrophages expressing CHOP were observed in the vehicle-treated pancreas in contrast to its PBA-treated counterpart (electronic supplementary material, figure S14). Furthermore, pancreatic islets in the PBA-treated allo-BMT recipient mice were larger than those in their vehicle-treated counterparts (figure 5*e*; electronic supplementary material, figure S15). Presumably, as a result of the protection of exocrine and endocrine glands, the blood glucose levels in the PBA-medicated allo-BMT recipients were normal in contrast to those in their vehicle-medicated equivalents (*p* = 2.2 × 10^−7^) (figure 5*f*). Immunoblot analysis of IL-6 and CTGF also indicated that systemic injection of PBA suppressed cGVHD-elicited inflammation and fibrosis in contrast to the solvent-vehicle (IL-6: *p* = 6.9 × 10^−7^, CTGF: *p* = 1.6 × 10^−8^) (electronic supplementary material, figures S16 and S17). Moreover, judging from electron micrographic analysis, pancreatic epithelia treated with PBA had almost normal structure of ER, blood vessels and mitochondria (figure 5*g*; electronic supplementary material, figure S18). By contrast, in vehicle-treated pancreatic epithelia, the ER appeared to be expanded due to abnormal accumulation of proteins, the wall of a capillary was extremely thin, and mitochondria seemed to be damaged and lose their cristae (figure 5*g*; electronic supplementary material, figure S18).

### 3.6. Repression of chronic graft-versus-host disease-elicited epithelial to mesenchymal transition in pancreas by 4-phenylbutyric acid

**Figure 6.**
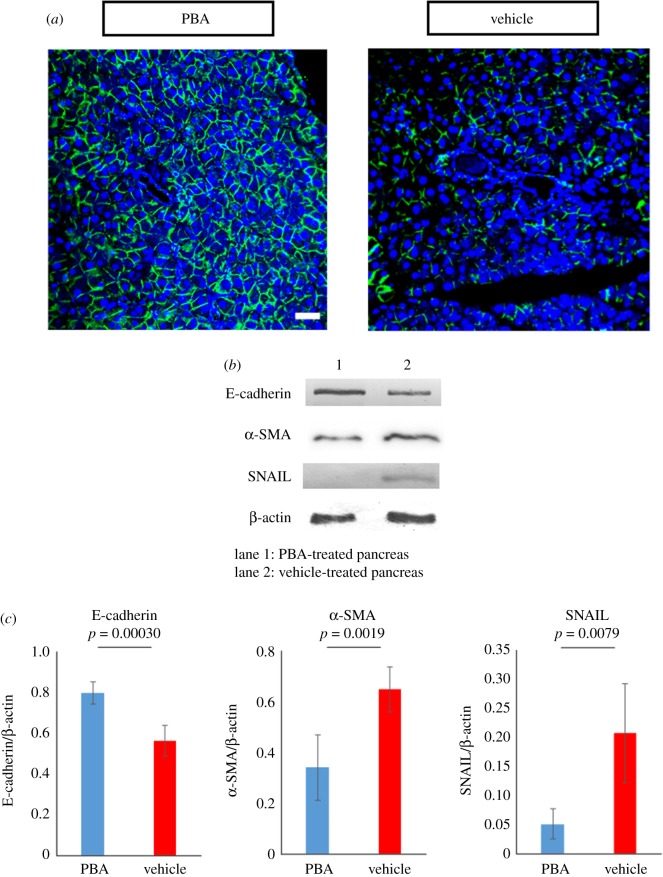
EMT markers in the cGVHD-affected pancreas. (*a*) Immunostaining for E-cadherin in the PBA-treated pancreas and its vehicle-treated counterpart. E-cadherin and cell nuclei are stained green and blue, respectively. The fluorescence images were at 200× magnification, and the scale bar is 20 µm. (*b*) Immunoblot analysis of E-cadherin and α-SMA was carried out (lane 1: PBA-injected pancreas, lane 2: vehicle-injected pancreas). Cropped blots are displayed, and the corresponding full-length gels are shown in electronic supplementary material, figure S19. (*c*) The corresponding quantitative analysis of the protein bands was conducted. PBA-medicated pancreas (blue) and its vehicle-medicated counterpart (red). Results are representative of two independently performed experiments with similar results. The data are presented as means ± s.d., PBA: *n* = 6, vehicle: *n* = 6.

We subsequently investigated whether PBA could suppress EMT, which was conceivably associated with excessive fibrosis in the pancreas affected by cGVHD. Immunohistochemistry and immunoblot analysis revealed that E-cadherin was retained in the PBA-treated pancreas in contrast to its vehicle-treated counterpart (*p* = 7.4 × 10^−4^) (figure 6*a*–*c*; electronic supplementary material, figure S19a). In addition, the protein levels of α-SMA and Snail in the PBA-medicated pancreas seemed to be normal, whereas α-SMA and Snail were overexpressed in its vehicle-medicated partner (α-SMA: *p* = 0.0019 and Snail: *p* = 0.0082) (figure 6*b*,*c*; electronic supplementary material, figure S19*b,c*).

## 4. Discussion

Allogeneic HSCT recipients are subjected to risk of cGVHD, which can affect multiple organs and cause a wide range of disabling symptoms. Although cGVHD in many organs has been investigated over recent decades, pancreatic cGVHD has been virtually unstudied. Thus, we were prompted to examine the state of the pancreas affected by cGVHD using a well-established mouse model. As demonstrated by our previous investigation, the pathogenic processes of lacrimal gland cGVHD in this murine model were similar to those in patients with cGVHD [3,35–37]. As with the human lacrimal glands affected by cGVHD, abnormal inflammation and fibrosis were observed in their murine counterparts. In addition, previous reports demonstrated that inflammation and fibrosis were induced systemically in this mouse model [3,36,38]. Therefore, we examined the development of pancreatic cGVHD using this murine model.

Our histological investigation revealed that the pancreas in allo-BMT recipient mice was aberrantly inflamed and fibrotic and that the pancreas was vulnerable to cGVHD. In particular, severe inflammation and fibrosis around pancreatic ducts were induced, and pancreatic islets were shrunk. Previous reports indicated that exocrine failure as well as endocrine failure in the pancreas could result in diabetes [25–27]. Hence, it is conceivable that the exocrine and endocrine failure in cGVHD-affected pancreas resulted in diabetes-like symptoms and thereby the blood glucose levels in mice with cGVHD were augmented. Moreover, as confirmed by immunoblot investigation, the expression of the inflammatory marker IL-6 [28] and the fibrotic indicator CTGF [29] was higher in the cGVHD-affected pancreas compared with its syngeneic control partner. IL-6 is conceivably involved in the differentiation of naive T cells into regulatory T cells or proinflammatory T cells [39,40]. Hence, IL-6 has captured interest from many researchers working on GVHD. Also, because CTGF is reported to be overexpressed in a wide range of fibrosis-associated diseases, it can indicate the degree of fibrosis in a reliable manner [41]. Furthermore, our electron microscopic examination into pancreas disordered by cGVHD suggested that (i) the ER in the cGVHD-affected epithelia was expanded owing to the accumulation of proteins, (ii) the blood vessels were seriously demolished and (iii) mitochondria in the epithelia were severely damaged. Our survey of previous articles revealed that mitochondria could be destroyed as a result of increased ER stress. Hence, the expanded ER and the damaged mitochondria were indicative of escalation of ER stress.

Accordingly, with the novel findings above, we next investigate pancreatic cGVHD more closely. Our recent report indicates that the elevation of ER stress in cGVHD-disordered organs is associated with abnormal inflammation and fibrosis [10]. Thus, we envisaged that it was also applicable to the pancreas. Our data suggested (i) that ER stress markers were expressed at higher level in the pancreas affected by cGVHD compared with its syngeneic control counterpart and (ii) that accordingly the cGVHD-impaired pancreas displayed the higher expression of the inflammation-associated molecules NF-κB and TXNIP. The elevation of TXNIP prompted us to carry out a survey of literature, and previous articles demonstrated that TXNIP was detrimentally correlated with EMT and that EMT was related to fibrosis elicited by cGVHD [42,43]. In this study, we focused on the following EMT markers: E-cadherin, α-SMA and Snail. Literature precedent suggests (i) that EMT can be promoted by loss of E-cadherin, which is expressed in epithelial cells, (ii) that the increased expression of α-SMA in vascular smooth muscle cells and myoepithelial cells is correlated with EMT and (iii) that Snail is presumed to suppress the expression of E-cadherin [30,44]. Our investigation into the EMT indicators suggested that EMT occurred in the cGVHD-affected pancreas and contributed to the development of cGVHD-triggered fibrosis in a deleterious manner.

With these outcomes, we subsequently attempted to treat pancreatic cGVHD. As reported in our recent article, the ER stress reducer PBA can be an effective medication to treat cGVHD in various organs. Thus, we attempted to combat pancreatic cGVHD by treating allo-BMT recipients with PBA. Our data suggested the ability of PBA to reduce cGVHD-induced ER stress and the corresponding proinflammatory molecules in the pancreas. As a consequence, judging from the results of histological and immunohistochemical examination, cGVHD-caused inflammation and fibrosis were reduced in the PBA-treated pancreas by contrast with its vehicle-treated partner. Especially, PBA treatment subdued inflammation and fibrosis around pancreatic ducts, prevented pancreatic islets from shrinking and retained normal blood glucose levels in all-BMT recipient mice. Electron microscopic assays also underpinned our claim by showing that PBA kept the ER, blood vessels and mitochondria in pancreatic epithelia virtually intact.

Literature precedent suggests that PBA shows the capacity to restrain EMT, which results in fibrosis [45]. This report and our encouraging outcomes prompted us to more meticulously investigate how PBA protected the pancreas from fibrosis induced by cGVHD. Our immunohistochemical and immunoblot assays for EMT makers in the PBA- and vehicle-injected pancreas suggested that PBA could suppress EMT induced by cGVHD. As stated above, TXNIP is reported to be implicated in EMT. Thus, it is conceivable that PBA suppressed the ER stress-induced expression of TXNIP, with the result that EMT caused by cGVHD was subdued.

In clinical settings, some cGVHD patients suffer from diabetes. Historically, it has been presumed to arise from the conventional steroid treatment [8,9]. However, our basic study has suggested that diabetes in allogeneic HSCT recipients could result from cGVHD-induced inflammation and fibrosis in the pancreas and that the ER stress attenuator PBA has the potential to prevent the new onset of diabetes.

Overall, our animal study has indicated that the pancreas can be disordered by cGVHD-elicited inflammation and fibrosis and that systemic injection of PBA can prevent and/or mitigate pancreatic cGVHD. Hence, our novel investigation into pancreatic cGVHD may change the current insights into diabetes occurring in allogeneic HSCT recipients and facilitate the treatment of cGVHD in medical settings.

## Supplementary Material

Supplementary Figures

## Supplementary Material

Data for Fig 2

## Supplementary Material

Data for Fig 4

## Date accessibility

The datasets supporting this article have been uploaded as part of the electronic supplementary material.
