# Supplementary material for: Novel elucidation and treatment of pancreatic chronic graft-versus-host disease in mice
Source: R Soc Open Sci. 2018 Oct 17;5(10):181067. doi: 10.1098/rsos.181067 (PMC6227968; doi:10.1098/rsos.181067)
Supplement: Supplementary Figures [file rsos181067supp1.doc]

**Novel Elucidation and Treatment of Pancreatic Chronic Graft-Versus-Host Disease in Mice**

Shin Mukai,^1, 3^ Yoko Ogawa,^1^ Fumihiko Urano,^2^ Yutaka Kawakami,^3^ and Kazuo Tsubota^1^

^1^Deaprtment of Ophthalmology, Keio University School of Medicine, ^2^Department of Medicine, Division of Endocrinology, Metabolism, and Lipid Research, and Department of Pathology and Immunology, Washington University School of Medicine, ^3^Institute for Advanced Medical Research, Keio University School of Medicine.

**Supplementary Figure 1**

**Supplementary Figure 1**. **The enlarged versions of the HE pictures of the pancreas collected from syngeneic control subjects and cGVHD-affected mice shown in Figure 1a.** The images were taken at 200x magnification, and the scale bar is 200 μm. Severely inflamed portions are shown with blue asterisks.

**Supplementary Figure 2**

**Supplementary Figure 2**. **The enlarged versions of the Mallory pictures of the pancreas collected from syngeneic control subjects and cGVHD-affected mice shown in Figure 1b.** The images were taken at 200x magnification, and the scale bar is 200 μm. Aberrantly fibrotic areas are shown with white asterisks.

**Supplementary Figure 3**

**Supplementary Figure 3**. **The enlarged versions of the fluorescence images of the pancreas collected from syngeneic control subjects and cGVHD-affected mice shown in Figure 1c.** The images were taken at 200x magnification, and the scale bar is 200 μm.

**Supplementary Figure 4**

**Supplementary Figure 4**. **The enlarged versions of the HE pictures of pancreatic islets in syngeneic control subjects and cGVHD-affected mice shown in Figure 1e.** The images were taken at 400x magnification, and the scale bar is 100 μm. A pancreatic islet is shown with a black arrow.

**Supplementary Figure 5**

**(a)**

**(b)**

**Supplementary Figure 5.** (a) Immunoblot analysis of IL-6 and CTGF was carried out. (Lane 1: Syngeneic control subject, Lane 2: cGVHD-affected pancreas) Cropped blots are displayed, and the corresponding full-length gels are shown in Supplementary Figure 6. (b) Densitometric analysis of the protein bands was subsequently conducted. cGVHD-affected pancreas (red) and its syngeneic control partner (blue). Data from one of two similar experiments are shown. The data are presented as means, ± SD, Control: n=5, cGVHD: n=5

**
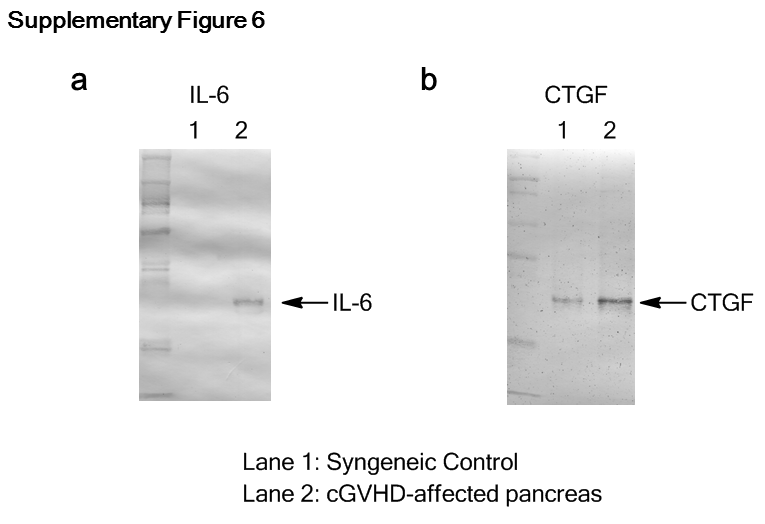
**

**Supplementary Figure 6. Full-length gels from immunoblot assays for IL-6 and CTGF shown in Supplementary Figure 5.** Lane 1: Syngeneic Control. Lanes 2: cGVHD-affected pancreas. (a) IL-6, (b) CTGF

**Supplementary Figure 7**

**Supplementary Figure 7**. **The enlarged versions of the electron micrographs of the pancreas collected from syngeneic control subjects and cGVHD-affected mice shown in Figure 1g.** Cap: Capillary, M: Mitochondrion. The pictures of stroma in the pancreas (left) were at 2000x magnification, and the scale bar is 5 μm. Asterisks are placed where the ER is expanded due to the accumulation of proteins, and cell debris is shown with rectangles. The photographs of blood vessels in the pancreas (middle) were at 5000x magnification, and the scale bar is 500 nm. Damaged blood vessels are displayed with an ellipse. The photographs of mitochondria in the pancreas (right) were at 15000x magnification, and the scale bar is 500 nm.

**
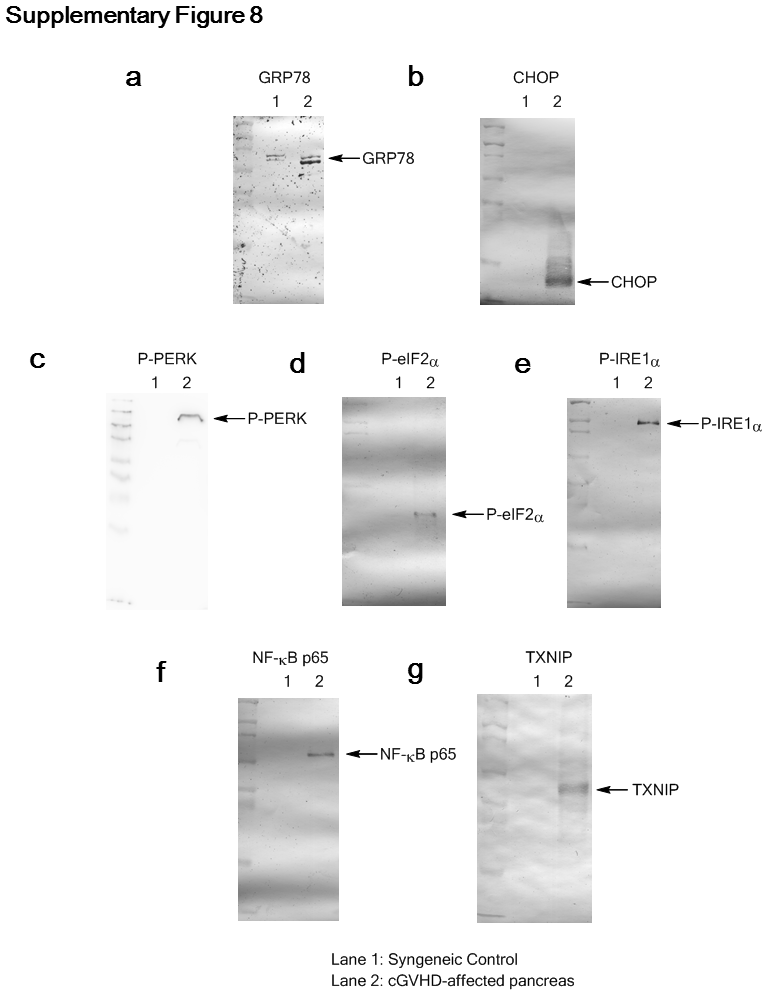
**

**Supplementary Figure 8. Full-length gels from immunoblot assays for markers of ER stress and inflammation-associated molecules shown in Figure 2a.** Lane 1: Syngeneic Control. Lanes 2: cGVHD-affected pancreas. (a) GRP78, (b) CHOP, (c) P-PERK, (d) P-eIF2α, (e) P-IRE1α, (f) NF-κB p65, (g) TXNIP

**
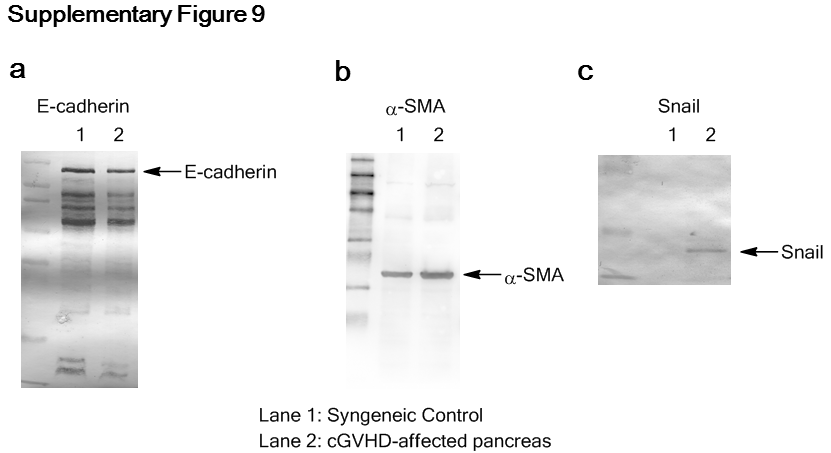
**

**Supplementary Figure 9. Full-length gels from immunoblot assays for EMT markers shown in Figure 3b.** Lane 1: Syngeneic Control. Lanes 2: cGVHD-affected pancreas. (a) E-cadherin, (b) α-SMA, (c) Snail

**
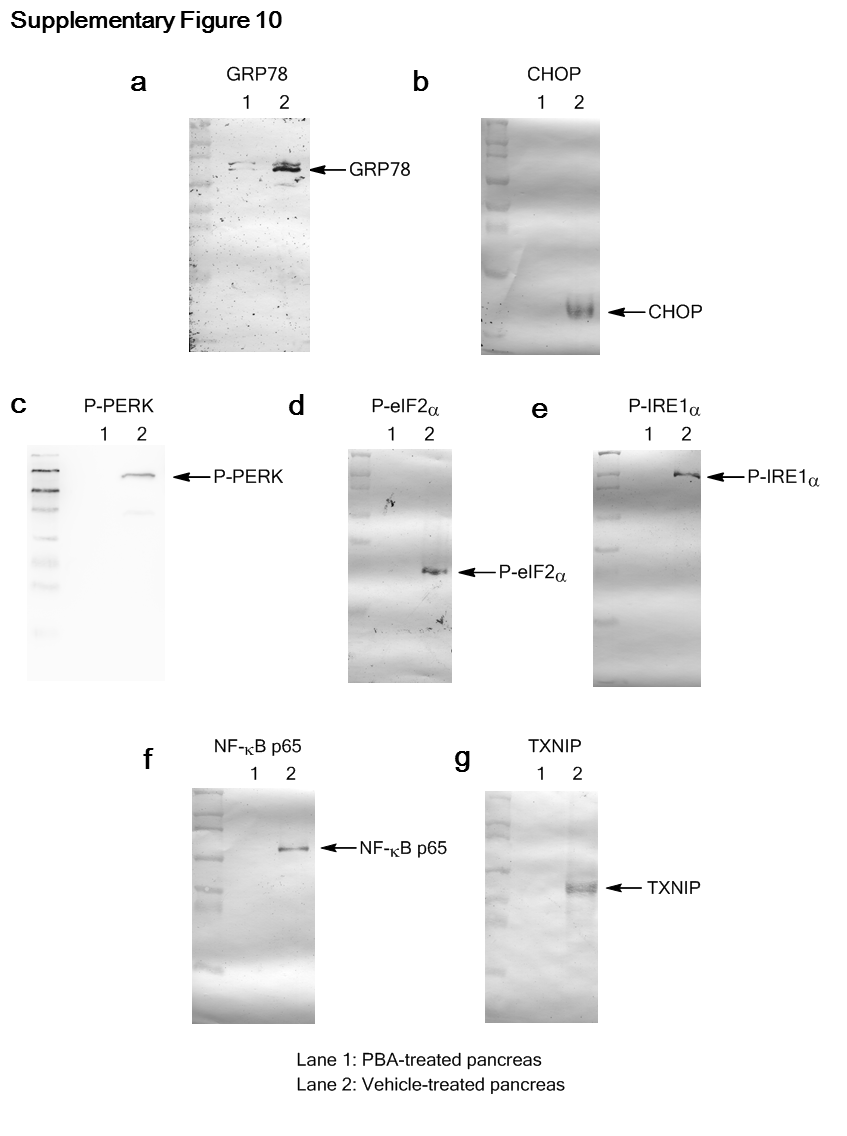
**

**Supplementary Figure 10. Full-length gels from immunoblot assays for markers of ER stress and inflammation-associated molecules shown in Figure 4a.** Lane 1: PBA-treated pancreas. Lanes 2: Vehicle-treated pancreas. (a) GRP78, (b) CHOP, (c) P-PERK, (d) P-eIF2α, (e) P-IRE1α, (f) NF-κB p65, (g) TXNIP

**Supplementary Figure 11**

**Supplementary Figure 11**. **The enlarged versions of the HE pictures of the pancreas collected from PBA- and vehicle-treated allo-BMT recipient mice shown in Figure 5a.** The images were taken at 200x magnification, and the scale bar is 200 μm. Severely inflamed portions are shown with blue asterisks.

**Supplementary Figure 12**

**Supplementary Figure 12**. **The enlarged versions of the Mallory pictures of the pancreas collected from PBA- and vehicle-treated allo-BMT recipient mice shown in Figure 5b.** The images were taken at 200x magnification, and the scale bar is 200 μm. Aberrantly fibrotic areas are shown with white asterisks.

**Supplementary Figure 13**

**Supplementary Figure 13**. **The enlarged versions of the fluorescence images of the pancreas collected from PBA- and vehicle-treated recipient mice shown in Figure 5c.** The images were taken at 200x magnification, and the scale bar is 200 μm.

**Supplementary Figure 14**

**Supplementary Figure 14.** Multiple staining for CHOP and CD68 in the pancreas collected from PBA- and vehicle-treated allo-BMT recipient mice. CHOP, CD68 and nuclei are stained green, red and blue, respectively. The images were taken at 200x magnification, and the scale bar is 20 μm.

**Supplementary Information 15**

**Supplementary Figure 15**. **The enlarged versions of the HE pictures of pancreatic islets in PBA- and vehicle-treated allo-BMT recipient mice shown in Figure 5e.** The images were taken at 400x magnification, and the scale bar is 100 μm. A pancreatic islet is shown with a black arrow.

**Supplementary Information 16**

**(a)**

**(b)**

**Supplementary Figure 16.** (a) Immunoblot analysis of IL-6 and CTGF was carried out. (Lane 1: PBA-medicated pancreas, Lane 2: Vehicle-medicated pancreas) Cropped blots are displayed, and the corresponding full-length gels are shown in **Supplementary Figure 17**. (b) Densitometric analysis of the protein bands was subsequently conducted. PBA-treated pancreas (blue) and its vehicle-treated partner (red). Results are representative of 2 independently performed experiments with similar results. The data are presented as means, ± SD, PBA: n=6, Vehicle: n=6


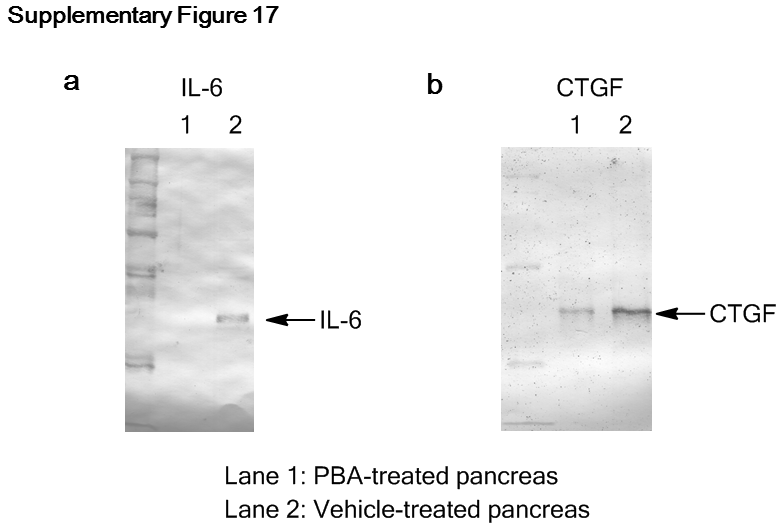


**Supplementary Figure 5. Full-length gels from immunoblot assays for IL-6 and CTGF shown in Supplementary Figure 16.** Lane 1: PBA-treated pancreas. Lanes 2: Vehicle-treated pancreas. (a) IL-6, (b) CTGF

**Supplementary Figure 18**

**
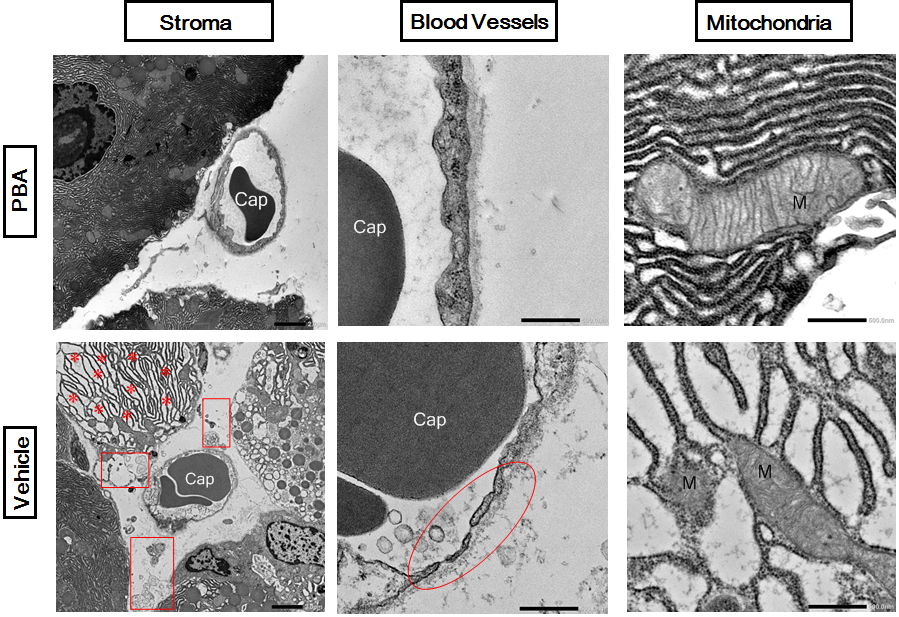
**

**Supplementary Figure 18**. **The enlarged versions of the electron micrographs of the pancreas collected from PBA- and vehicle-treated allo-BMT recipient mice shown in Figure 5g.** Cap: Capillary, M: Mitochondrion. The pictures of stroma in the pancreas (left) were at 2000x magnification, and the scale bar is 5 μm. Asterisks are placed where the ER is expanded due to the accumulation of proteins, and cell debris is shown with rectangles. The photographs of blood vessels in the pancreas (middle) were at 5000x magnification, and the scale bar is 500 nm. Damaged blood vessels are displayed with an ellipse. The photographs of mitochondria in the pancreas (right) were at 15000x magnification, and the scale bar is 500 nm.

**
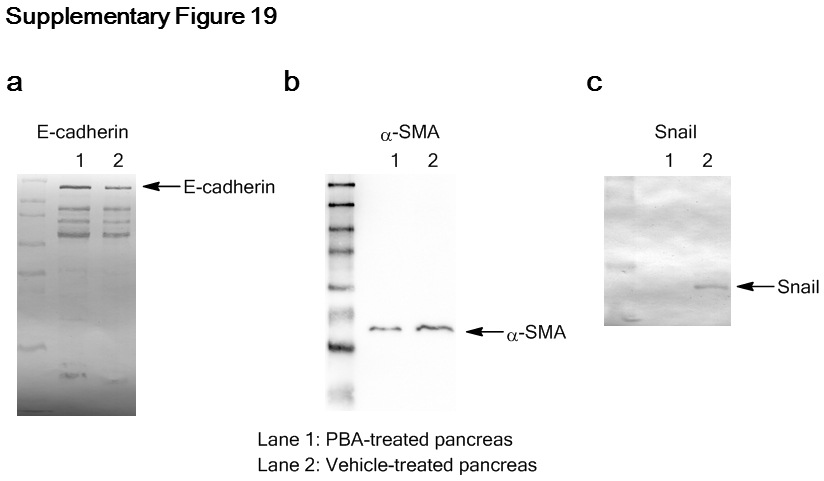
**

**Supplementary Figure 19. Full-length gels from immunoblot assays for EMT markers shown in Figure 6b.** Lane 1: PBA-treated pancreas. Lanes 2: Vehicle-treated pancreas. (a) E-cadherin, (b) α-SMA, (c) Snail
