# Supplementary material for: Novel elucidation and treatment of pancreatic chronic graft-versus-host disease in mice
Source: R Soc Open Sci. 2018 Oct 17;5(10):181067. doi: 10.1098/rsos.181067 (PMC6227968; doi:10.1098/rsos.181067)
Supplement: Data for Fig 2 [file rsos181067supp2.zip › How to see the raw data (cGVHD vs Control).doc]

**How to see the raw data (cGVHD [n=5] vs Control [n=5])**

Densitometry XXX in Pancreas (cGVHD vs Control) Raw Data- 1

1. Syngeneic Control 1

2. cGVHD 1

3. Syngeneic Control 2

4. cGVHD 2

5. Syngeneic Control 3

6. cGVHD 3

Densitometry XXX in Pancreas (cGVHD vs Control) Raw Data- 2

1. Syngeneic Control 4

2. cGVHD 4

3. Syngeneic Control 5

4. cGVHD 5
