# Supplementary material for: Novel elucidation and treatment of pancreatic chronic graft-versus-host disease in mice
Source: R Soc Open Sci. 2018 Oct 17;5(10):181067. doi: 10.1098/rsos.181067 (PMC6227968; doi:10.1098/rsos.181067)
Supplement: Data for Fig 4 [file rsos181067supp3.zip › How to see the raw data (PBA vs Vehicle).doc]

**How to see the raw data (PBA [n=6] vs Vehicle [n=6])**

Densitometry XXX in Pancreas (PBA vs Vehicle) Raw Data- 1

1. PBA 1

2. Vehicle 1

3. PBA 2

4. Vehicle 2

5. PBA 3

6. Vehicle 3

Densitometry XXX in Pancreas (PBA vs Vehicle) Raw Data- 2

1. PBA 4

2. Vehicle 4

3. PBA 5

4. Vehicle 5

5. PBA 6

6. Vehicle 6
